# Protective Effects of Oroxylin A against Doxorubicin-Induced Cardiotoxicity via the Activation of Sirt1 in Mice

**DOI:** 10.1155/2021/6610543

**Published:** 2021-01-19

**Authors:** Wen-Bin Zhang, Yong-Fa Zheng, Yao-Gui Wu

**Affiliations:** Department of Oncology, Renmin Hospital of Wuhan University, Wuhan, Hubei 430060, China

## Abstract

Doxorubicin- (DOX-) related cardiac injury impairs the life quality of patients with cancer. This largely limited the clinical use of DOX. It is of great significance to find a novel strategy to reduce DOX-related cardiac injury. Oroxylin A (OA) has been identified to exert beneficial effects against inflammatory diseases and cancers. Here, we investigated whether OA could attenuate DOX-induced acute cardiotoxicity in mice. A single dose of DOX was used to induce acute cardiac injury in mice. To explore the protective effects, OA was administered to mice for ten days beginning from five days before DOX injection. The data in our study indicated that OA inhibited DOX-induced heart weight loss, reduction in cardiac function, and the elevation in myocardial injury markers. DOX injection resulted in increased oxidative damage, inflammation accumulation, and myocardial apoptosis in vivo and in vitro, and these pathological alterations were alleviated by treatment of OA. OA activated the sirtuin 1 (Sirt1) signaling pathway via the cAMP/protein kinase A, and its protective effects were blocked by Sirt1 deficiency. OA treatment did not affect the tumor-killing action of DOX in tumor-bearing mice. In conclusion, OA protected against DOX-related acute cardiac injury via the regulation of Sirt1.

## 1. Introduction

Doxorubicin (DOX), a quinone-containing anthracycline, is used to treat leukemia and malignant lymphomas. The use of DOX triggered toxic effects on the hearts and resulted in the cardiomyocyte loss and congestive heart failure, which limited the clinical use of DOX [1, 2]. DOX-induced characteristic change of acute cardiotoxicity included myofibrillar disruption, cardiomyocytes atrophy, and vacuolated preapoptotic cells, while cumulative chronic cardiotoxicity could lead to ventricular dilation [3]. Previous studies had identified the mammalian target of rapamycin (mTOR) as a key determinant of DOX-related cardiotoxicity [4–6]. However, there were no drugs that could effectively prevent the toxic effects of DOX. Accumulating evidences suggested that DOX-induced myocardial injury may be related to oxidative stress, calcium overload, mitochondrial damage, and cardiomyocyte apoptosis [7].

It has been reported that the metabolic products of DOX could transfer its unpaired electrons to oxygen, and thus, inducing the production of free radicals and cardiotoxicity [8]. In addition, the early activation of nuclear factor kappa-B (NF-*κ*B) and subsequent inflammatory factors accumulation were also involved in DOX-related cardiac injury [9]. Oxidative stress caused the release of cytochrome c and the increased caspase-3 activity, promoting myocardial apoptosis [10]. Therefore, it is important to find a novel drug that constrains these pathological alterations for the treatment of DOX-related myocardial injury.

Oroxylin A, a natural flavonoid extracted from Scutellaria radix, has been reported to exert anticancer activities by inhibiting tumor invasion and metastasis [11]. The anticolon cancer effects of OA were associated with the inhibition of the inflammatory response [12, 13]. OA could also inhibit hypoxia-inducible factor-1 (HIF-1) *α* signaling pathway in mice [14]. In addition, OA treatment inhibited hydrogen peroxide-induced oxidative damage of PC12 cells [15] and attenuated oxidative stress caused by cigarette smoke via activating nuclear factor- (erythroid-derived 2-) like 2 (Nrf2) signaling pathway [16]. However, the effects of OA on DOX-induced acute cardiotoxicity and the related signaling mechanisms have not yet been reported. In the present study, we show that OA protects the mice against DOX-induced acute cardiotoxicity by activating sirtuin 1 (Sirt1) signaling pathways. Our studies suggest that OA might have therapeutic utility in the treatment of DOX-induced myocardial injury.

## 2. Materials and Methods

### 2.1. Reagents

Oroxylin A (purity > 98% as determined by HPLC) was purchased from the National Institutes for Food and Drug Control (China). The specific Sirt1 inhibitors including EX527 (HY-15452) and nicotinamide (HY-B0150) were provided by MedChemExpress (Shanghai, China). DOX was provided by Sigma-Aldrich (St. Louis, MO, USA). The assay kits for Sirt1 activity (fluorometric, ab156065), NAD/NADH level (colorimetric, ab65348), protein kinase A (PKA, ab139435) activity, and cAMP level (competitive ELISA, ab138880) were provided by Abcam (Cambridge, UK).

### 2.2. Animals and Treatment

All animals' experimental protocols were approved by the Ethical Committee of Renmin Hospital of Wuhan University. The male C57BL/6 mice (age: 8-10 weeks, body weight: 23.5-27.5 g) were purchased from the animal experiment center of Wuhan University (Wuhan, China). All the mice were housed in the specific-pathogen-free mouse room of Renmin Hospital of Wuhan University under the standard conditions. Mice were randomly assigned into four groups as control+vehicle, control+OA, DOX+vehicle, and DOX+OA (*n* = 10 per group). The mice in our study were intraperitoneally injected with DOX at a dose of 20 mg/kg to establish the model of DOX-related cardiac injury [17]. To evaluate the effects of OA on DOX-induced acute cardiotoxicity, mice were orally given OA (40 mg/kg) or the same volume of CMC-Na solution for ten days beginning from five days before DOX injection. The dose of OA referred to a previous study [18]. Five days after DOX, the mice were anesthetized with isoflurane, and blood was collected via retro-orbital sinus for further detection. To confirm the role of Sirt1 in the protection of OA treatment, cardiac-restricted Sirt1 knockout (cKO) mice were used [19]. In brief, Sirt1 conditional floxed mice were bred with mice carrying the *α*-Mhc-MerCreMer transgene (Jackson Laboratory) to generate cardiac-restricted Sirt1 cKO mice. To delete Sirt1, Sirt1 cKO mice were intraperitoneally injected with tamoxifen (25 mg/kg/day) for 5 consecutive days.

In the tumor experiment, Lewis lung carcinoma (LLC) cells were subcutaneously implanted to isogenic mice. OA was administered on day 5 after tumor implantation, and this protective intervention lasted for ten days. DOX was injected on day 10. Five days after DOX, the mice were anesthetized with isoflurane, and blood and hearts were collected for further detection [20].

### 2.3. Hemodynamics

Left ventricle hemodynamics were detected as previously described [21]. Mice were anesthetized, and the apex of the left ventricle was exposed. After that, a micronanometer catheter (Millar 1.4F, SPR 835, Millar Instruments, TX, USA) was inserted into the left ventricle. This transducer was connected to a Power Laboratory system to detect and analyze the obtained data.

### 2.4. Detection of Myocardial Injury Markers

Five days after DOX, the mice were anesthetized with isoflurane, and blood was collected via retro-orbital sinus. The lactate dehydrogenase (LDH) detection kit (C0017) was provided by Beyotime Biotechnology (Beijing, China). And the creatine kinase myocardial bound (CK-MB) detection kit (E006-1-1) was provided by Nanjing Jiancheng Bioengineering Institute (Nanjing, China). The homogenate of hearts and blood supernatants was used to measure the two myocardial injury markers.

### 2.5. Assessment of Biochemical Parameters

To detect myocardial oxidative injury, fresh heart samples were collected. The levels of myocardial 4-hydroxynonenal (4-HNE), nitrotyrosine, and glutathione (GSH) as well as the activities of glutathione peroxidase (Gpx) and superoxide dismutase (SOD) were detected following the manufacturer's instructions. 4-HNE ELISA Kit (E4645-100), nitrotyrosine ELISA Kit (K4158-100), and GSH Assay Kit (K264-100) were provided by BioVision (San Francisco, USA). Gpx BioAssay ELISA Kit (#356081) was also provided by BioVision. The activity of total SOD was detected using a kit provided by Nanjing Jiancheng Bioengineering Institute (Nanjing, China).

### 2.6. Cardiomyocyte Isolation and Contractile Assay

We used a Langendorff perfusion system to isolate adult cardiomyocytes from the hearts of DOX-treated mice according to a previous study [22]. Cardiomyocyte contractile function was detected using a SoftEdge MyoCam® system, which was obtained from IonOptix Corporation (MA, USA). Resting cell length, peak shortening, maximal velocity of shortening (+dL/dt), and maximal velocity of relengthening (-dL/dt) were detected.

### 2.7. Western Blot

After, the heart samples were collected and the total proteins were extracted using a BCA protein assay kit (Thermo Scientific, Rockford, USA). Nuclear protein was extracted using NE-PER™ Nuclear Extraction Reagents (Thermo Fisher Scientific). And then, the samples were separated by 10% SDS-PAGE and transferred to PVDF membranes [23, 24]. The membranes were then incubated with the primary antibodies against Nrf2 (Abcam, ab62352, 1 : 1000), heme oxygenase-1 (HO-1, Abcam, ab68477, 1 : 1000), NADPH quinone acceptor oxidoreductase 1 (NQO1, Abcam, ab28947, 1 : 1000), GAPDH (Abcam, ab8245, 1 : 1000), inhibitor-*κ*B*α* (Abcam, ab32041, I*κ*B*α*, 1 : 1000), P-I*κ*B*α* (Abcam, ab38515, 1 : 1000), NF-*κ*B (Abcam, ab16502, 1 : 1000), Histone H3 (Abcam, ab1791, 1 : 1000), Bcl-2 (Abcam, ab32124, 1 : 500), and Sirt1 (Abcam, ab189494, 1 : 1000) followed by the incubation of the secondary antibodies. These primary antibodies were obtained from Abcam (Cambridge, UK). These bands were detected by Bio-Rad imaging system and quantified by the Image Lab Software.

### 2.8. Quantitative Real-Time PCR

Total RNAs were isolated from heart tissues using the TRIzol reagent (Invitrogen Life Technologies, USA) [25]. cDNA synthesis was performed with the Bio-Rad iScript™ cDNA synthesis kit. The mRNA levels of genes were determined using the Transcriptor First Strand cDNA Synthesis kit (Roche, Germany). GAPDH was used as the reference gene.

### 2.9. Apoptotic Assay

The hearts were fixed with 4% paraformaldehyde for 24 hours. After that, the hearts were subjected to the standard procedures and then were sliced into sections. TUNEL staining was performed with a kit (Millipore, Billerica, MA, USA) following the manufacturer's instructions. Myocardial apoptosis was also assayed by the detection of caspase3/7 activity and poly ADP-ribose polymerase (PARP) activity.

### 2.10. Cell Culture

H9c2 cell line was purchased from the Institute of Biochemistry Cell Biology (Shanghai, China), and the cells were maintained in DMEM supplemented with 10% fetal bovine serum [26]. The H9c2 cells were seeded in 96-well plates at a density of 5 × 10^4^ cells/mL for 24 h and pretreated with 40 *μ*mol/l of OA for 24 h before challenged with DOX (5 *μ*mol/l) for 24 h. The dose of OA was determined according to a previous study [27]. Cell viability was detected by a CCK-8 kit. Intracellular hydrogen peroxide was detected by an Intracellular Hydrogen Peroxide Detection Kit (BioVision, #K204-200), and superoxide was detected by a kit called Superoxide Anion Assay Kit (Sigma, CS1000). To verify the hypothesis that OA provided protection via activation of Sirt1, cells were subjected to EX527 (1 *μ*mol/l) or nicotinamide (100 *μ*mol/l) at 1 hour before DOX administration. NF-*κ*B DNA binding activity and Nrf2 DNA binding activity were detected by the kits called TransAM® NF*κ*B and TransAM® Nrf2 (Active motif, USA). To explore the mechanism by which OA treatment activated Sirt1, cells were incubated with H89 (a PKA inhibitor, 10 *μ*mol/l), 2′5′-dd-Ado (an adenylate cyclase inhibitor, 200 *μ*mol/L) for 24 hours.

### 2.11. Detection of Cellular ROS

H9c2 myocytes were cultured in 96-well plates and pretreated with OA and DOX for 24 hours. 2,7-Dichlorofluorescin diacetate (DCFH-DA) was obtained from Nanjing Jiancheng Bioengineering Institute (Nanjing, China). Reactive oxygen species (ROS) were then detected by DCFH-DA. The cells were incubated with DCFH-DA (10 *μ*mol/L) for 2 hours at 37°C, and immunofluorescence was detected by a fluorescence microplate reader as described previously [26].

### 2.12. Statistical Analysis

All the data were presented as the mean ± standard deviation (SD). We used unpaired Student's *t*-test to compare differences between the two groups. Differences between multiple groups were determined by one-way ANOVA followed by Tukey's test. *P* < 0.05 was considered as significant.

## 3. Results

### 3.1. OA Suppressed Cardiac Injury and Attenuated Cardiac Dysfunction in DOX-Induced Mice

**Figure 1:**
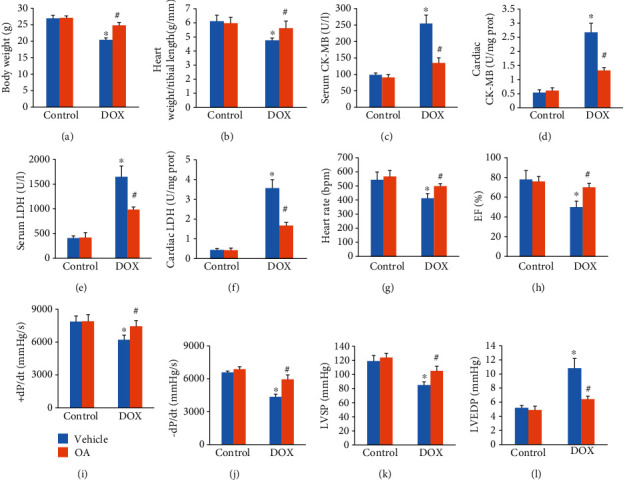
OA treatment attenuated cardiac dysfunction in DOX-treated mice. (a) Bodyweight in the indicated group (*n* = 10). (b) The ratio of heart weight to tibial length (*n* = 10). (c, d) Serum and cardiac CK-MB (*n* = 6). (e, f) Serum and cardiac LDH (*n* = 6). (g) Heart rate in the indicated groups (*n* = 6). (h) EF in the indicated groups (*n* = 6). (i, j) ±dP/dt in DOX-treated mice (*n* = 6). (k, l) LVSP and LVEDP in DOX-treated mice (*n* = 6). Differences between multiple groups were determined by one-way ANOVA followed by Tukey's test. ^∗^*P* < 0.05 vs. control group, ^#^*P* < 0.05 vs. DOX group.

Mice in the DOX group exhibited lower body weight (BW) and ratio of heart weight to tibial length (HW/TL) than those of the control group (Figures 1(a) and 1(b)). Compared to the DOX group, BW and HW/TL in the OA-treated group were significantly increased (Figures 1(a) and 1(b)). Next, we detected CK-MB and LDH, which are regarded as markers of cardiac injury. The increased plasma and cardiac CK-MB were significantly suppressed by OA treatment in DOX-treated mice (Figures 1(c) and 1(d)). The data in our study also suggested that DOX significantly increased the levels of LDH in the hearts and plasma, which were largely reduced by the treatment of OA (Figures 1(e) and 1(f)). Heart rate, ejection fraction (EF), maximum first derivative of ventricular pressure with respect to time (+dP/dt), -dP/dt, and left ventricle systolic pressure (LVSP) were significantly reduced in the DOX group. However, these alterations were largely attenuated by OA treatment (Figures 1(g)–1(k)). In addition, OA treatment suppressed the elevation of left ventricular end-diastolic pressure (LVEDP) in DOX-treated mice (Figure 1(l)).

### 3.2. OA Improved Contractile Function in Cardiomyocytes Isolated from DOX-Treated Mice

**Figure 2:**
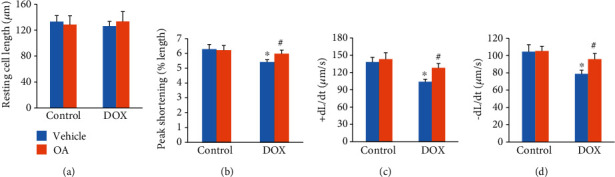
OA treatment improved contractile function in cardiomyocytes. (a) Resting cell length (*n* = 6). (b) Peak shortening (*n* = 6). (c, d) ±dL/dt (*n* = 6). Differences between multiple groups were determined by one-way ANOVA followed by Tukey's test. ∗*P* < 0.05 vs. control group, ^#^*P* < 0.05 vs. DOX group.

DOX injection did not affect the resting cell length of the isolated cardiomyocytes (Figure 2(a)). In response to DOX injection, cardiomyocytes isolated from DOX-treated mice showed decreased peak shortening, +dL/dt, and -dL/dt. And these pathological alterations were largely prevented by the treatment of OA (Figures 2(b)–2(d)).

### 3.3. OA Treatment Attenuated DOX-Induced Oxidative Damage in Cardiac Tissues

**Figure 3:**
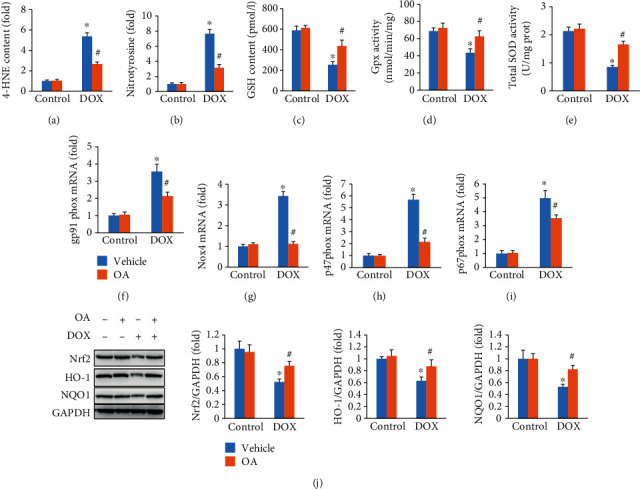
OA treatment prevented oxidative damage induced by DOX in cardiac tissue. (a, b) Myocardial 4-HNE and nitrotyrosine (*n* = 6). (c) GSH levels (*n* = 6). (c, d) Gpx and SOD activity (*n* = 6). (f–i) The mRNA levels of gp91phox, Nox4, p47phox, and p67phox (*n* = 6). (j) The protein expression of Nrf2 and downstream targets (*n* = 6). Differences between multiple groups were determined by one-way ANOVA followed by Tukey's test. ^∗^*P* < 0.05 vs. control group, ^#^*P* < 0.05 vs. DOX group.

To investigate the effects of OA treatment on oxidative stress, we first detected the products of lipid peroxidation. In response to DOX injection, myocardial 4-HNE and nitrotyrosine levels were significantly increased. And these alterations were largely attenuated by OA treatment (Figures 3(a) and 3(b)). Administration of DOX decreased the content of GSH, Gpx, and total SOD activities in the hearts, and OA treatment prevented these pathological changes caused by DOX injection (Figures 3(c)–3(e)). Further detection revealed that the increased mRNA expression of gp91phox, NADPH oxidase 4, p47phox, and p67phox in DOX-treated mice was largely suppressed by OA treatment (Figures 3(f)–3(i)). The expression levels of Nrf2 and the downstream HO-1 and NQO1 were significantly decreased. In contrast, after OA treatment, these decreased protein expressions were significantly reversed in DOX-injected mice (Figure 3(j)).

### 3.4. OA Attenuated the Upregulation of Inflammatory Cytokines and Myocardial Apoptosis in DOX-Treated Mice

**Figure 4:**
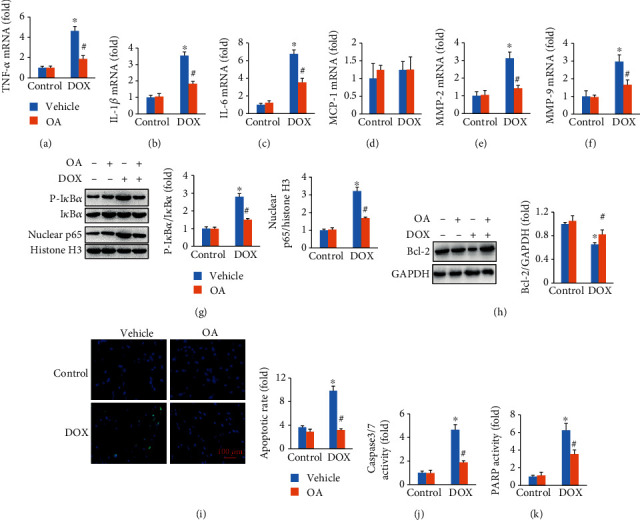
OA treatment attenuated inflammatory response and myocardial apoptosis in DOX-treated hearts. (a–d) Relative mRNA levels of inflammatory factors (*n* = 6). (e, f) The mRNA levels of MMP-2 and MMP-9 (*n* = 6). (g) The protein expression of p-I*κ*B*α* and nuclear p65 (*n* = 6). (h) The protein expression of Bcl-2 (*n* = 6). (i) TUNEL staining and apoptotic rate (*n* = 6). (j, k) Caspase3/7 and PARP activities (*n* = 6). Differences between multiple groups were determined by one-way ANOVA followed by Tukey's test. ^∗^*P* < 0.05 vs. control group, ^#^*P* < 0.05 vs. DOX group.

As shown in Figures 4(a)–4(c), DOX increased the mRNA levels of tumor necrosis factor-*α* (TNF-*α*), interleukin-6 (IL-6), and IL-1*β*, which were remarkably inhibited by OA treatment (Figures 4(a)–4(c)). Unexpectedly, there was no difference between the four groups about the expression of monocyte chemotactic protein 1 (MCP-1) (Figure 4(d)). Inflammation induced the activation of matrix metalloproteinases (MMPs), which were closely involved in the pathogenesis of DOX-related cardiac injury [28]. Thus, we detected the mRNA levels of MMP2 and MMP-9 and found that the increased expression of MMP-2 and MMP-9 was suppressed by OA treatment (Figures 4(e) and 4(f)). NF-*κ*B acted as a transcriptional factor and was responsible for the expression of several inflammatory cytokines including TNF-*α* and IL-6. The phosphorylation of I*κ*B*α* protein played a key role in the activation of NF-*κ*B [29]. Interestingly, DOX treatment significantly increased the phosphorylation of I*κ*B*α* protein, and this alteration was blocked by OA (Figure 4(g)). In agreement with this finding, we also found that DOX increased the nuclear translocation of NF-*κ*B, and this effect was attenuated by the treatment of OA (Figure 4(g)). Next, we assessed the antiapoptotic activity of OA. The decreased Bcl-2 protein expression caused by DOX injection was reversed by the treatment of OA (Figure 4(h)). Compared with the control group, DOX notably induced apoptosis in the hearts, as detected by TUNEL staining, caspase3/7 activity, and PARP activity. OA treatment attenuated these pathological alterations (Figures 4(i)–4(k)).

### 3.5. OA Treatment Inhibited DOX-Induced Cardiomyocytes Injury via Activating Sirt1

**Figure 5:**
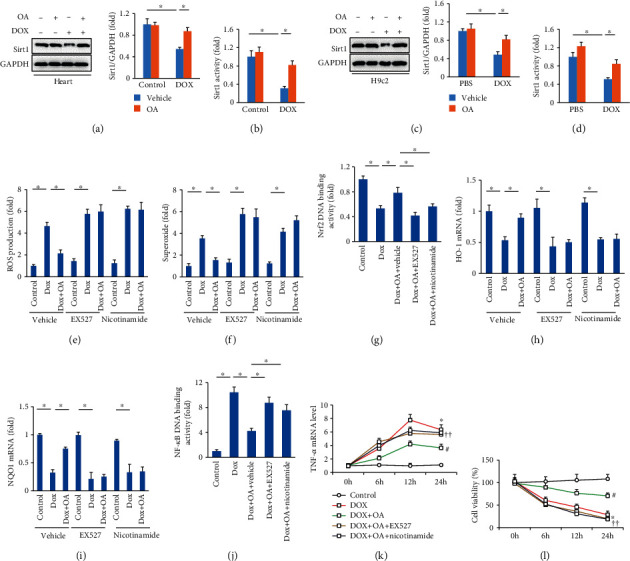
OA treatment exerted protection via Sirt1. (a, b) The Sirt1 protein expression and activity in vivo (*n* = 6). (c, d) The Sirt1 protein expression and activity in vitro (*n* = 6). (e, f) The production of ROS and superoxide (*n* = 5). (g) Nrf2 DNA binding activity (*n* = 5). (h, i) The mRNA levels of HO-1 and NQO1 (*n* = 6). (j) NF-*κ*B binding activity (*n* = 5). (k) The mRNA level of TNF-*α* (*n* = 5). (l) Cell viability (*n* = 5). Differences between multiple groups were determined by one-way ANOVA followed by Tukey's test. For (a–i), ^∗^*P* < 0.05 vs. matched control; for (j, k), ^∗^*P* < 0.05 vs. control group, ^#^*P* < 0.05 vs. DOX group, ^†^*P* < 0.05 vs. DOX+OA group.

Previous studies suggested that Sirt1 could inhibit NF-*κ*B [30]. Thus, we determined whether OA activated Sirt1 in vivo and in vitro. As shown in Figures 5(a) and 5(b), DOX decreased myocardial Sirt1 protein expression and activity, and these reductions were significantly normalized by OA treatment. In line with the findings in vivo, the decreased protein expression and activity of Sirt1 were also significantly restored in OA-treated H9c2 cells (Figures 5(c) and 5(d)). To confirm the role of Sirt1 in OA-mediated protection of DOX-related cardiac injury, we used the specific Sirt1 inhibitor EX527 and nicotinamide. We found that OA treatment significantly decreased the production of ROS and superoxide in DOX-treated cells, and these protection of OA against ROS and superoxide production were abolished by the use of EX527 or nicotinamide (Figures 5(e) and 5(f)). Next, we used a kit to detect Nrf2 DNA binding activity and found that OA enhanced Nrf2 transcriptional activity in response to DOX and lost this ability after Sirt1 inhibition by EX527 or nicotinamide (Figure 5(g)). The subsequent detection of the mRNA levels of HO-1 and NQO1 revealed that OA lost its effects on the expression of HO-1 and NQO1 after Sirt1 inhibition (Figures 5(h) and 5(i)). OA inhibited NF-*κ*B transcriptional activity in response to DOX and lost this ability after Sirt1 inhibition by EX527 or nicotinamide (Figure 5(j)). EX527 or nicotinamide also abolished the inhibitory effects exhibited by OA treatment on the mRNA level of TNF-*α* in DOX-treated cells (Figure 5(k)). Cell viability was decreased in the DOX-treated group, increased after OA treatment, but declined again after Sirt1 inhibition by EX527 or nicotinamide (Figure 5(l)).

### 3.6. OA Lost the Protective Effects in Sirt1-Deficient Mice

**Figure 6:**
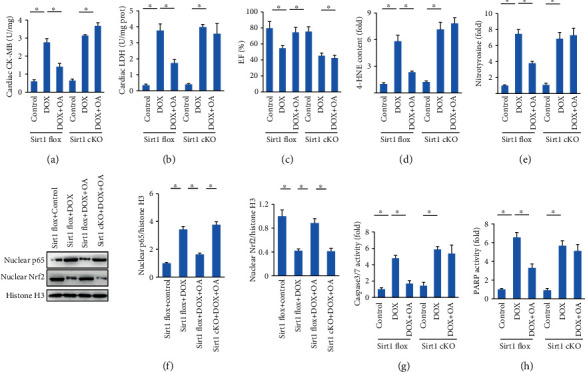
Sirt1 depletion abolished the protection provided by OA. (a, b) The cardiac CK-MB and LDH in DOX-treated mice (*n* = 6). (c) EF in the indicated groups (*n* = 10). (d, e) Myocardial 4-HNE and nitrotyrosine (*n* = 6). (f) The protein expression of nuclear Nrf2 and NF-*κ*B (*n* = 6). (g, h) Caspase3/7 and PARP activities (*n* = 6). Differences between multiple groups were determined by one-way ANOVA followed by Tukey's test. ^∗^*P* < 0.05 vs. matched control.

Subsequently, we determined whether OA treatment lost its protective effects on DOX-related cardiac injury when Sirt1 signaling was blocked. To achieve this, we used mice with a cardiac-specific deletion of Sirt1 (Sirt1 cKO). Interestingly, OA treatment in the absence of Sirt1 did not attenuate DOX-induced cardiac injury. Instead, OA-treated Sirt1 cKO mice exhibited a similar phenotype as that in Sirt1 cKO mice in response to DOX injection, as reflected by cardiac CK-MB and LDH, cardiac function, 4-HNE, and nitrotyrosine (Figures 6(a)–6(e)). The inhibitory effect of OA nuclear translocation of NF-*κ*B was also blocked by Sirt1 deficiency. The increased nuclear Nrf2 expression after OA treatment was also suppressed by Sirt1 deficiency (Figure 6(f)). The subsequent detection of the activities of caspase3/7 and PARP revealed that Sirt1 depletion also abolished the antiapoptotic effects of OA (Figures 6(g) and 6(h)).

### 3.7. OA Treatment Activated Sirt1 via cAMP/PKA in Mice

**Figure 7:**
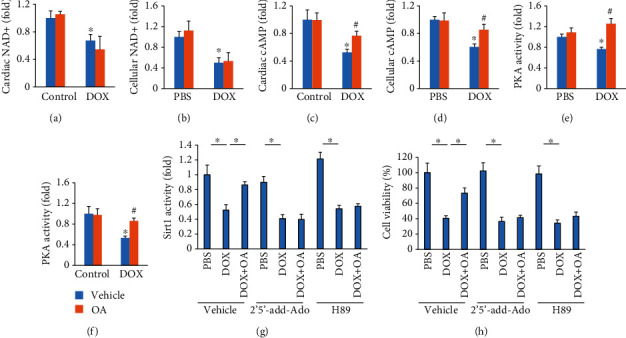
OA treatment activated Sirt1 via cAMP/PKA signaling axis. (a, b) Relative NAD+ levels (*n* = 6). (c, d) Relative cAMP levels (*n* = 6). (e) Relative PKA activity in cells (*n* = 6). (f) Relative PKA activity in the hearts (*n* = 6). (g) Relative Sirt1 activity in cells (*n* = 6). (h) Cell viability of cells (*n* = 6). Differences between multiple groups were determined by one-way ANOVA followed by Tukey's test. For (a–e), ^∗^*P* < 0.05 vs. control/PBS group, ^#^*P* < 0.05 vs. DOX group. For others, ^∗^*P* < 0.05 versus the matched group.

Sirt1 is a well-known NAD+-dependent deacetylase, and the NAD+ concentrations can affect Sirt1 activation. DOX decreased cardiac and cellular NAD+ levels, surprisingly, OA treatment could not affect NAD+ levels in DOX-treated hearts or cells (Figures 7(a) and 7(b)). Interestingly, we found OA treatment increased cAMP abundance in DOX-treated hearts and cells (Figures 7(c) and 7(d)). The decreased PKA activity in DOX-treated hearts and cells was restored by OA treatment (Figures 7(e) and 7(f)). Moreover, Sirt1 activation by OA treatment was blunted in cells with an adenylate cyclase inhibitor (2′5′-dd-Ado) or a PKA specific inhibitor (H89) treatment (Figure 7(g)). OA lost the protective effects on cell viability in DOX-treated cells with adenylate cyclase or PKA inhibition (Figure 7(h)).

### 3.8. OA Treatment Did Not Affect Tumor Growth or Tissue Concentrations of DOX

**Figure 8:**
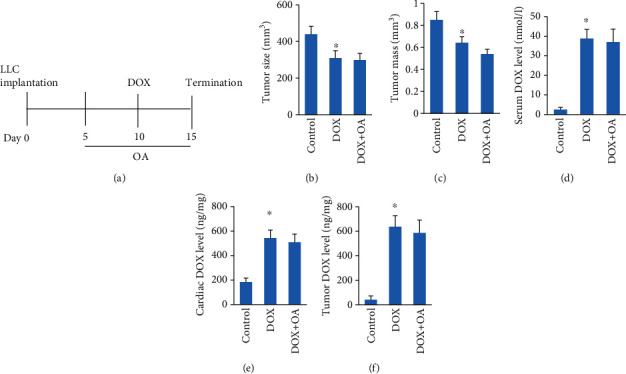
OA treatment did not increase tumor growth. (a) Schedule of the experiment. (b, c) Tumor size and tumor tissue mass (*n* = 6). (d–f) DOX concentrations in vivo in tissue (*n* = 6). Differences between multiple groups were determined by one-way ANOVA followed by Tukey's test. ^∗^*P* < 0.05 vs. matched control.

Next, we evaluated whether OA treatment affected tumor growth or tissue concentrations of DOX. Lewis lung carcinoma (LLC) cells were subcutaneously implanted to isogenic C57Bl6 mice. OA was administered on day 5 after tumor implantation, and this protective intervention lasted for ten days. DOX was injected on day 10 (Figure 8(a)). The data in our study indicated that OA treatment did not affect the growth of LLC xenotransplants (Figures 8(b) and 8(c)). Next, we detected DOX concentrations in serum, heart, and tumor tissue (Figures 8(d) and 8(f)). We found that there were no difference between DOX and DOX+OA groups, implying that the protective effects of OA were not attributed to reduce the availability of DOX.

## 4. Discussion

Here, we showed that OA treatment suppressed DOX-induced cardiotoxicity, as indicated by the improved cardiac function, reduced oxidative damage, inflammatory response, and myocardial apoptosis. Furthermore, we found that these protective effects of OA were mediated by the activation of Sirt1 in vivo and in vitro, and Sirt1 inhibition abolished OA treatment-mediated cardiac protection. In addition, we found that OA treatment did not affect tumor growth and compromise the effects of DOX. Collectively, our data define OA as a potential therapeutic drug for DOX-induced cardiotoxicity.

Some lines of evidence have suggested that oxidative stress was closely involved in the pathogenesis of DOX-induced myocardial damage [31, 32]. DOX-related toxicity was mainly caused by the free radicals during DOX metabolism, which impaired mitochondrial respiratory complex and promoted the production of superoxide [33]. Heart samples are much more sensitive to DOX-related redox imbalance for the lack of redox cycle-related enzymes and the high volume of mitochondria [32]. Several lines highlighted the importance of modulating reactive oxidative damage in ameliorating the cardiotoxic effects caused by DOX. Nitric oxide synthase 3 deficiency could reduce the production of free radicals, thus, preventing the decline in cardiac function in DOX-treated mice [34]. Conversely, overexpression of SOD significantly decreased the oxidative damage and improved cardiac function in mice treated with DOX [35]. Previous studies found that OA could block free radical production in alcoholic liver disease [36]. In agreement with these findings, we also found that DOX impaired both SOD and Gpx activities, decreased GSH levels but increased lipid peroxidation contents, and these toxic effects of DOX were largely blocked by OA treatment. We also found that OA increased Nrf2 expression in DOX-treated mice. The attenuation of oxidative damage in OA-treated hearts might partly explain the protective effects of OA.

A number of studies suggested that the inflammatory response mediated the pathogenesis of DOX-induced heart dysfunction [37]. Upon stimuli, I*κ*B*α* was phosphorylated and became degraded, and NF-*κ*B translocated into the nucleus to trigger inflammatory cytokine synthesis [38]. Wang et al. first observed the activation of NF-*κ*B in DOX-treated myocytes [39]. Here, we also found that treatment with OA attenuated the DOX-induced activation of NF-*κ*B and upregulation of inflammatory factors. DOX-induced NF-*κ*B activation was closely associated with myocardial apoptosis [40]. Here, we also found that DOX could induce myocardial apoptosis in vivo and impair cell viability in vitro. OA decreased myocardial apoptosis and improved cell viability in response to DOX. The attenuation of inflammatory response and myocardial apoptosis also contributed to the protective effects of OA.

Sirt1 has been suggested to play key roles in redox regulation, cell apoptosis, and inflammation [41]. Several lines of evidences suggested that Sirt1 was also involved in DOX-induced cardiac injury. A study found Sirt1 protein level was increased in response to DOX injection [42]. Inconsistent with this finding, the data from another lab indicated that DOX induced a significant decrease in Sirt1 expression. Here, we also found that DOX decreased Sirt1 protein level in vivo and in vitro. Of note, restoration of the expression of Sirt1 by OA treatment could improve cardiac function and attenuate DOX-related cardiac injury in mice. Moreover, Sirt1 depletion offsets the protective effects provided by OA treatment against DOX-induced cardiotoxicity. This finding suggested that OA exerted cardiac protection via Sirt1.

OA treatment did not alter the NAD+ level, suggesting that OA activated Sirt1 through a NAD+ independent manner. cAMP serves as an important second messenger to activate Sirt1 in NAD+ independent ways [43, 44]. cAMP/PKA induced the dissociation of Sirt1 with its endogenous inhibitor [44]. Moreover, it has been reported that PKA promoted the phosphorylation of Sirt1 to increase its enzymatic activity [43]. Here, we found that OA treatment increased cAMP levels and PKA activity in DOX-treated cells. Moreover, inhibition of adenylate cyclase and PKA abolished the activation of Sirt1 by OA, indicating that cAMP/PKA was required for the activation of Sirt1 by OA treatment.

To enhance the translational potential of our findings, we next confirmed that OA treatment did not compromise therapeutic DOX levels or promote tumor growth. Actually, several studies have suggested that OA suppressed the development and growth of tumor [14]. Here, we found that OA treatment did affect tumor growth or tissue concentrations of DOX, implying that OA could not compromise therapeutic DOX levels.

In conclusion, mice treated with OA treatment revealed a suppressed myocardial toxicity, induced by DOX injection. OA may be considered as the new drug for the treatment of DOX-induced cardiotoxicity.

## Data Availability

The data in our study are available from the corresponding author upon reasonable request.
